# The effect of maternal BMI, smoking and alcohol on congenital heart diseases: a Mendelian randomisation study

**DOI:** 10.1186/s12916-023-02731-y

**Published:** 2023-02-01

**Authors:** Kurt Taylor, Robyn E. Wootton, Qian Yang, Sam Oddie, John Wright, Tiffany C. Yang, Maria Magnus, Ole A. Andreassen, Maria Carolina Borges, Massimo Caputo, Deborah A. Lawlor

**Affiliations:** 1 Bristol Medical School, Population Health Science, Bristol, BS8 2BN UK; 2 MRC Integrative Epidemiology Unit, University of Bristol, Bristol, BS8 2BN UK; 3 Nic Waals Institute, Lovisenberg Diaconal Hospital, Oslo, Norway; 4 University of York, Heslington, York UK; 5 Bradford Institute for Health Research, Bradford Teaching Hospitals NHS Foundation Trust, Bradford, UK; 6 Centre for Fertility and Health, Norwegian Institute of Public Health, Oslo, Norway; 7 Division of Mental Health and Addiction, NORMENT Centre, Oslo University Hospital and Institute of Clinical Medicine, University of Oslo, Oslo, Norway; 8 KG Jebsen Centre for Neurodevelopmental Disorders, Oslo University Hospital and Institute of Clinical Medicine, Oslo, Norway; 9 Bristol Medical School, Translational Science, Bristol, UK

**Keywords:** Congenital heart disease, Mendelian randomisation, Risk factors, ALSPAC, BiB, MoBa

## Abstract

**Background:**

Congenital heart diseases (CHDs) remain a significant cause of infant morbidity and mortality. Epidemiological studies have explored maternal risk factors for offspring CHDs, but few have used genetic epidemiology methods to improve causal inference.

**Methods:**

Three birth cohorts, including 65,510 mother/offspring pairs (*N* = 562 CHD cases) were included. We used Mendelian randomisation (MR) analyses to explore the effects of genetically predicted maternal body mass index (BMI), smoking and alcohol on offspring CHDs. We generated genetic risk scores (GRS) using summary data from large-scale genome-wide association studies (GWAS) and validated the strength and relevance of the genetic instrument for exposure levels during pregnancy. Logistic regression was used to estimate the odds ratio (OR) of CHD per 1 standard deviation (SD) higher GRS. Results for the three cohorts were combined using random-effects meta-analyses. We performed several sensitivity analyses including multivariable MR to check the robustness of our findings.

**Results:**

The GRSs associated with the exposures during pregnancy in all three cohorts. The associations of the GRS for maternal BMI with offspring CHD (pooled OR (95% confidence interval) per 1SD higher GRS: 0.95 (0.88, 1.03)), lifetime smoking (pooled OR: 1.01 (0.93, 1.09)) and alcoholic drinks per week (pooled OR: 1.06 (0.98, 1.15)) were close to the null. Sensitivity analyses yielded similar results.

**Conclusions:**

Our results do not provide robust evidence of an effect of maternal BMI, smoking or alcohol on offspring CHDs. However, results were imprecise. Our findings need to be replicated, and highlight the need for more and larger studies with maternal and offspring genotype and offspring CHD data.

**Supplementary Information:**

The online version contains supplementary material available at 10.1186/s12916-023-02731-y.

## Background

Congenital heart diseases (CHDs) are the most common congenital anomaly, affecting around 1% of live births and 10% of stillbirths [1, 2]. CHDs are a leading cause of childhood mortality and many CHD patients experience health problems that persist into adulthood [3, 4]. The causes of CHDs are largely unknown, but the pregnancy environment (intrauterine factors) may play a role in the underlying pathophysiology [5]. Identifying modifiable risk factors for CHDs is important for improving aetiological understanding and developing preventive interventions to reduce disease burden.

Several modifiable maternal characteristics have been found to be associated with increased risk of CHDs, including maternal pre/early pregnancy body mass index (BMI) [6–8], smoking [9] and alcohol [10] consumption in pregnancy. The causal relevance of the results from meta-analyses is unclear, due to many studies not controlling for key confounders and for the risk of residual confounding. Previously, using parental negative exposure control analyses, we found that positive associations between maternal overweight and obesity with offspring CHDs may be being driven by confounding factors [11]. This work found some evidence of an intrauterine effect of maternal smoking on offspring CHDs. For alcohol consumption, results were inconclusive due to limited data [11]. Negative control analyses attempt to address the issue of residual confounding in observational studies [6, 11, 12], but rely on assumptions that cannot be empirically verified, such as it being implausible that the exposure in the father (e.g. their smoking) could influence offspring CHD risk to a similar magnitude of any effect in mothers.

Mendelian randomisation (MR) uses genetic variants as instrumental variables (IVs) to test causal effects in observational data [13]. The key assumptions for MR are (i) relevance assumption—the genetic instruments are robustly associated with the exposure, (ii) independence assumption—there is no confounding of the genetic instrument-outcome association, (iii) exclusion restriction criteria—the genetic variant is not related to the outcome other than via its association with the exposure [14]. Genetic variants are less likely to be confounded by the socioeconomic and environmental factors that might bias causal estimates in conventional multivariable regression [15], but may be biased by violation of their assumptions due to weak or irrelevant instruments, population stratification (causing confounding of the genetic instrument-outcome association) and a path from the genetic instrument to CHD not mediated by the exposure, for example via horizontal pleiotropy or foetal genotype [16]. Triangulating results from negative control and MR analyses, whereby the key sources of bias differ can help improve the causal understanding of maternal risk factors on CHDs [17]. Consistent results from both would increase confidence that the relationship is causal. The recent acquisition of genotype information on a large number of maternal-offspring dyads means that we now have relevant data to further test the potential effects of BMI, smoking and alcohol with a complementary method to those used previously. The objective of this study was therefore to explore associations between genetically predicted maternal BMI, smoking and alcohol on offspring CHD using Mendelian randomisation.

## Methods

This study is reported using the Strengthening The Reporting of Observational Studies in Epidemiology Using Mendelian Randomisation (STROBE-MR) guidelines (see Additional File 1: STROBE-MR Checklist) [18, 19].

### Inclusion criteria and participating cohorts

**Fig. 1:**
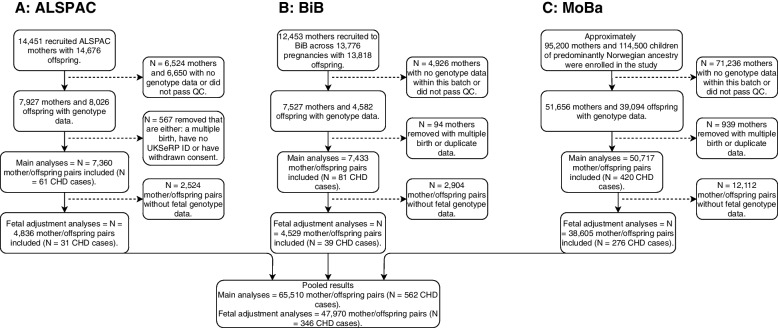
An overview of included cohorts and selection of study participants. Abbreviations: ALSPAC, Avon Longitudinal Study of Parents and Children; BiB, Born in Bradford; MoBa, Norwegian Mother, Father and Child Cohort; QC, quality control; UKSeRP, the secure research platform containing CHD data for ALSPAC; CHD, congenital heart disease; QC, quality control

To be eligible for inclusion in this study, cohorts and participants were required to have genome-wide data in mothers and CHD data in the offspring. From previous work with large consortia, including MR-PREG [20] and LifeCycle [21], we identified three cohorts meeting these criteria: The Avon Longitudinal Study of Parents and Children (ALSPAC), Born in Bradford cohort (BiB), and the Norwegian Mother, Father and Child Cohort Study (MoBa). ALSPAC is a UK prospective birth cohort study which was devised to investigate the environmental and genetic factors of health and development [22–24]. ALSPAC enrolled pregnant women who resided in and around the city of Bristol in the Southwest of England and had an expected delivery date between April 1, 1991, and December 31, 1992. The enrolled cohort included 15,247 pregnancies resulting in 14,775 live-born babies. Ethical approval was obtained from the ALSPAC Ethics and Law Committee and the Local Research Ethics Committees. The study website contains details of all the data that are available through a fully searchable data dictionary and variable search tool. BiB is a population-based prospective birth cohort including 12,453 women across 13,776 pregnancies who were recruited at their oral glucose tolerance test at approximately 26–28 weeks’ gestation [25]. Eligible women had an expected delivery between March 2007 and December 2010. MoBa is a nationwide, pregnancy cohort comprising family triads (mother-father-offspring) who are followed longitudinally. All pregnant women in Norway who were able to read Norwegian were eligible for participation. The first child was born in October 1999 and the last in July 2009 [26, 27]. One singleton pregnancy per mother in each cohort was included in analyses. Figure 1 shows the inclusion of participants, after excluding those with missing maternal genotype data and those that did not pass genetic quality control (QC). A total of 65,510 mother–offspring pairs contributed to the main analyses and 47,970 to the adjusted (for foetal genotype) analyses.

### Genetic data

#### Genotyping in each cohort

ALSPAC mothers were genotyped using Illumina human660K quad single nucleotide polymorphism (SNP) chip, and ALSPAC children were genotyped using Illumina HumanHap550 quad genome-wide SNP genotyping platform. Genotype data for both ALSPAC mothers and children were imputed against the Haplotype Reference Consortium v1.1 reference panel, after performing the QC procedure (minor allele frequency (MAF) ≥ 1%, a call rate ≥ 95%, in Hardy–Weinberg equilibrium (HWE), correct sex assignment, no evidence of cryptic relatedness, and of European descent). The samples of the BiB cohort (mothers and offspring) were processed on three different type of Illumina chips: HumanCoreExome12v1.0, HumanCoreExome12v1.1 and HumanCoreExome24v1.0. Genotype data were imputed against UK10K + 1000 Genomes reference panel, after a similar QC procedure (a call rate ≥ 99.5%, correct sex assignment, no evidence of cryptic relatedness, correct ethnicity assignment). In MoBa, blood samples were obtained from both parents during pregnancy and from mothers and children (umbilical cord) at birth [28]. Genotyping has had to rely on several projects—each contributing with resources to genotype subsets of MoBa over the last decade. The data used in the present study was derived from a cohort of genotype samples from four MoBa batches. The MoBa genetics QC procedure involved MAF ≥ 1%, a call rate ≥ 95%, in HWE, correct sex assignment, and no evidence of cryptic relatedness. Further details of the genotyping methods for each cohort are provided in the Supplementary Material (Additional File 2: Text S1).

#### GWAS data and SNP selection

We selected SNPs from the largest and most relevant GWAS of European ancestry participants for each exposure (further information for each GWAS is shown in Additional File 2: Table S1). Selected SNPs were those with a p-value below a *p*-value threshold used to indicate genome-wide significance after accounting for multiple testing. Of those reaching this threshold we ensured that we only took forward independent SNPs to create the GRSs (described below). For BMI, there were 941 near-independent SNPs in a combined GWAS of ~ 700,000 individuals as reported in Yengo et al. [29] (near-independent SNPs defined as SNPs with a *P* < 1 × 10^−8^ after a conditional and joint multiple SNP analysis to take into account linkage disequilibrium (LD) between SNPs at a given locus). For smoking analyses, there were 126 independent SNPs (genome-wide significant (*p* < 5 × 10^−8^) SNPs that achieved independence at LD r^2^ = 0.001 and a distance of 10,000 kb). The study was a GWAS of a lifetime smoking index (which combined smoking initiation, duration, heaviness and cessation), conducted in a sample of 462,690 current, former and never smokers in UK Biobank [30]. A GRS based on the lifetime smoking GWAS has previously been shown to be associated with smoking behaviours during pregnancy in the ALSPAC cohort [31]. For the alcohol weighted GRS, there were 99 conditionally independent SNPs (*P* < 5 × 10^−8^), measured as the number of alcoholic drinks per week [32]. This GRS has also previously been shown to be associated with alcohol consumption during pregnancy as well as the general population [33]. The ALSPAC cohort was included within the original GWAS for alcohol by Liu et al., accounting for 8,913 participants out of a total sample size of 941,280 (0.9%). Previous work has suggested any bias introduced by this level of overlap would be minimal [34]. Furthermore, a recent study explored this by excluding ALSPAC from the summary statistics and the results were unbiased and largely unchanged [33]. Therefore, we proceeded to use the full summary data for generating the alcohol GRS. All GRSs were generated using summary GWAS data that was derived in both men and women. We were unable to obtain female-specific summary data for these GWAS data. However, we performed checks to ensure the GRSs are robustly associated with the maternal exposure during pregnancy.

#### Genetic risk score generation

Weighted GRSs were calculated for BMI, smoking and alcohol consumption by adding up the number of risk factor increasing alleles among the selected SNPs after weighting each SNP by its effect on the corresponding risk factor:

$$\mathrm{GRS}=w1 \times \mathrm{SNP}1+w2 \times \mathrm{SNP}2+\dots wn \times \mathrm{SNP}n$$

where w is the weight (i.e. the beta-coefficient for the SNP-exposure association reported from the published GWAS) and SNP is the genotype dosage of exposure-increasing alleles at that locus (i.e. 0, 1, or 2 exposure-increasing alleles). Selected SNPs were extracted from the imputed genotype data in dosage format using QCTOOL (v2.0) and VCF tools (v 0.1.12b) in ALSPAC and BiB, respectively. PLINK (v1.9) was then used to construct the GRS for each exposure coded so that an increased score was associated with increased exposure. In MoBa, we constructed the GRSs from the QC’d data in PLINK format. Further information on GRS construction for each cohort is shown in Additional File 2: Text S2 [35, 36].

### Phenotype data

#### CHD data

In the ALSPAC cohort, cases were obtained from a range of data sources, including health record linkage and questionnaire data up until age 25 following European surveillance of congenital anomalies (EUROCAT) guidelines [37]. In BiB, cases were identified from either the Yorkshire and Humber congenital anomaly register database, which will tend to pick up most cases that were diagnosed antenatally and in the early postnatal period of life, and through linkage to primary care (up until aged 5), which will have picked up any additional cases, in particular those that might have been less severe and not identified antenatally/in early life [38]. All these cases were confirmed postnatally and were assigned international classification of disease Version 10 (ICD-10) codes. ICD-10 codes were used to assign CHD cases according to EUROCAT guidelines. In MoBa, information on whether a child had a CHD or not (yes/no) was obtained through linkage to the Medical Birth Registry of Norway (MBRN). All maternity units in Norway must notify births to the MBRN, and information on malformations is reported to the registry up to 12 months postpartum [39]. Further details on defining CHDs including ICD codes used (in ALSPAC and BiB) are shown in Additional File 2: Text S3 and Table S2.

#### Pregnancy phenotype data

As noted above, the SNP selection and weights for the GRS were taken from GWAS in women and men [29, 30, 32]. To determine their relevance in women during pregnancy we examined the associations of the GRS with pre/early pregnancy BMI, and pregnancy smoking and alcohol consumption in each cohort. In ALSPAC and MoBa, pre-pregnancy weight and height were self-reported during the first pregnancy questionnaires. In BiB, weight and height were measured at the recruitment assessment. As the timing of questions and the details requested for smoking during pregnancy differed across the three cohorts [40–42] we were only able to generate a simple binary variable of any smoking in pregnancy versus none. There was insufficient data and/or power across the cohorts to be able to generate a measure of smoking heaviness in pregnancy. As with smoking, the aim for alcohol was to determine whether the GRS was robustly associated with drinking status during pregnancy. We used questionnaire data in each cohort and used binary variables (yes/no) for whether women consumed any alcohol during pregnancy or not. Further details regarding these phenotype data, including questionnaire information and how these variables were derived, are described in Additional File 2: Text S4.

### Statistical analysis

Analyses were performed in R version 4.0.2 (R Foundation for Statistical Computing, Vienna, Austria). A pre-specified analysis plan was uploaded to the Open Science Framework [43]. We undertook MR in each of the 3 cohorts, including all ALSPAC, BiB and MoBa participants, with maternal genetic data and offspring CHD data. Logistic regression was used to estimate the odds ratio (OR) of CHD per 1 standard deviation (SD) higher GRS, with adjustment for the first 10 genetic principal components (PCs) with additional adjustment for genetic chip, genetic batch, and imputation batch in MoBa.

The key assumptions of MR are (i) relevance assumption, (ii) independence assumption and (iii) exclusion restriction. To explore the relevance of the GRS to each exposure in pregnancy, we undertook linear (BMI) and logistic (smoking and alcohol) regression to derive the difference in mean BMI and OR of pregnancy smoking and pregnancy alcohol consumption per 1SD higher GRS in each cohort. For BMI, instrument strength was assessed with F-statistics and *R*^2^. For smoking and alcohol, instrument strength was assessed using the area under the ROC curve and pseudo-*R*^2^ by the Nagelkerke method [44]. To minimise the potential for confounding of the GRS-CHD association due to population stratification, we adjusted for the first 10 ancestry-informative PCs [45]. We also repeated the MR analyses without the inclusion of BiB, given that BiB has a unique ethnic structure of South Asians and White Europeans. To explore horizontal pleiotropy, we checked the association of GRSs with known risk factors for CHD that we had data on (education, parity and diabetes), and as we were hypothesising that BMI, smoking and alcohol caused CHD we also explored the association of each GRS with the other two exposures. These analyses were performed using linear or logistic regression. Information on how these risk factors were assessed in each cohort is provided in the Supplementary Material (Additional File 2: Text S4). If any of the GRSs were associated with another risk factor, we considered that a potential horizontal pleiotropic effect. We then performed multivariable MR (MVMR) analyses if GWAS data for the potential horizontal pleiotropic variable was available (with the exception BMI and diabetes as it is on the causal path from BMI (rather than a separate path)) [46]. Methods for these GRSs and the rationale for selecting these risk factors are described in Additional File 2: Text S5 [47–54]. In sensitivity analyses to explore potential violation of the exclusion restriction criteria (IV assumption 3) via foetal genotype we repeated the PC (and batch) adjusted GRS-CHD association in the subsample of participants with foetal genome-wide data (Fig. 1) and then compared those results with the same associations additionally adjusted for the foetal GRS. GRS-CHD association results were pooled using a random effects meta-analysis for all three cohorts and fixed-effect meta-analyses when excluding BiB in sensitivity analyses (i.e. ALSPAC and MoBa). Between study heterogeneity was assessed using the Cochrane Q-statistic and I [2, 55].

## Results

### Participant characteristics

Analyses included 65,510 mother–offspring pairs, of which 562 offspring had CHD (Fig. 1). The distributions of offspring and maternal characteristics for these analyses in ALSPAC, BiB and MoBa are displayed in Table 1. The prevalence of any CHD, mean maternal age and pre-/early-pregnancy BMI were similar in the three cohorts. Women in ALSPAC were more likely to smoke during pregnancy in comparison to those in BiB and MoBa although, the overall prevalence in BiB masks marked differences between the two largest ancestral groups, with 3.4% of South Asian women reporting smoking during pregnancy compared to 34% of White European women. Women in ALSPAC and BiB were more likely to consume alcohol compared to those in MoBa, although, in BiB, there are limited data available on alcohol consumption with very few South Asians responding to questions relating to alcohol in questionnaires.

**Table 1:** Participant characteristics for the 3 studies included in Mendelian randomisation analyses

Characteristic	Category	ALSPAC (*N* = 7360)	BiB (*N* = 7433)	MoBa (*N* = 50,717)
***Offspring***				
CHD	Yes	61 (0.8)	81 (1.1)	420 (0.8)
Sex	Male	3703 (50.3)	3818 (51.4)	25,729 (51.0)
	Female	3657 (49.7)	3615 (48.6)	24,810 (49.0)
***Maternal***				
Age, years		29.2 (4.6)	27.4 (5.6)	30.2 (4.6)
Parity	Primiparous	3257 (46.6)	2963 (40.1)	27,199 (53.6)
BMI, kg/m^2^		22.5 (4.2)	26.2 (5.7)	24.1 (4.3)
Ethnicity	White European	7360 (100.0) ^a^	3084 (42.6)	50,717 (100.0) ^b^
	South Asian	-	3503 (48.4)	-
	Other	-	656 (9.1)	-
Any smoking during pregnancy	Yes	1679 (26.1)	1175 (18.1)	4175 (10.1)
Any alcohol during pregnancy	Yes	4866 (79.9)	1040 (49.3)	12,602 (32.6)

Data are means ± SD or n (%) unless stated. % are based on data available (data were not complete)

*Abbreviations*: *BiB*, Born in Bradford; *ALSPAC*, Avon Longitudinal Study of Parents and Children; *MoBa*, Norwegian Mother, Father and Child Cohort Study; *CHD*, congenital heart disease; *BMI*, body mass index; *kg*, kilogrammes; *m*, metres

^a^All non-white European women with ethnicity data were not included in the analysis

^b^Individuals of non-European ancestries were removed based on principal component analysis

### MR results

There were similar statistically positive associations of the BMI GRS with pre-pregnancy BMI and the smoking GRS with pregnancy smoking in all three cohorts (Table 2). The alcohol GRS also associated positively with alcohol consumption during pregnancy in all three cohorts with a somewhat weaker association in BiB and MoBa in comparison to ALSPAC. R^2^ and F-statistics for BMI suggested strong instruments, whereas for smoking and alcohol in particular the AUC suggested possible weak instruments.

**Table 2:** Relevance and strength of the genetic risk scores with exposures in pregnancy

Study	*N* participants	N SNPs in GRS	Coefficient (95% CI)^a^	*P*-value	*R*^2^/pseudo R^2 b^	F statistic ^c^	AUC
**Association of GRS for BMI with pre-/early-pregnancy BMI**							
ALSPAC	6253	941	0.24 (0.21, 0.26)	1 × 10^−80^	5.6%	372	-
BiB	6196	939	0.20 (0.18, 0.23)	5 × 10^−59^	4.1%	268	-
MoBa	45,033	868	0.25 (0.24, 0.26)	< 1 × 10^−100^	6.3%	3,023	-
**Association of GRS for a lifetime smoking index with any smoking during pregnancy**							
ALSPAC	6428	126	1.27 (1.20, 1.35)	1 × 10^−16^	1.6%	-	0.56
BiB	6482	126	1.36 (1.27, 1.45)	2 × 10^−20^	2.2%	-	0.59
MoBa	41,292	119	1.27 (1.23, 1.31)	5 × 10^−47^	1.0%	-	0.57
**Association of GRS for drinks per week with any alcohol consumption in pregnancy**							
ALSPAC	6087	98	1.14 (1.07, 1.21)	3 × 10^−5^	0.4%	-	0.53
BiB	2110	99	1.08 (0.99, 1.18)	0.09	0.2%	-	0.52
MoBa	38,645	73	1.02 (0.99, 1.04)	0.17	0.007%	-	0.50
MoBa sensitivity ^d^	38,645	73	1.05 (1.02, 1.08)	0.003	0.04%	-	0.51

*Abbreviations: SNP* Single nucleotide polymorphism, *GRS* Genetic risk score, *CI* Confidence interval, *AUC* Area under the curve, *ALSPAC* Avon Longitudinal Study of Parents and Children, *BiB* Born in Bradford, *MoBa* Norwegian Mother, Father and Child Cohort

^a^Effect estimates (coefficient) are difference in mean (BMI) or odds ratio (smoking or drinking yes/no during pregnancy) per SD increase in genetic risk score

^b^For the binary outcomes (smoking and alcohol) pseudo-*R*^2^ are presented

^c^For BMI F-statistic is presented; for binary outcomes (smoking and alcohol) AUC is presented

^d^In MoBa 32% consumed any alcohol during pregnancy. However, 63% of those consumed alcohol “less than once per month” based on the questionnaire data. In the sensitivity analysis shown above, we re-coded the variable so that those that consumed alcohol less than once per month were classed as non-drinkers (N.B. due to the small numbers in each individual category, we were not able to analyse these separately). This was performed as an additional check to ensure the GRS was associated with pregnancy alcohol consumption in MoBa

**Fig. 2:**
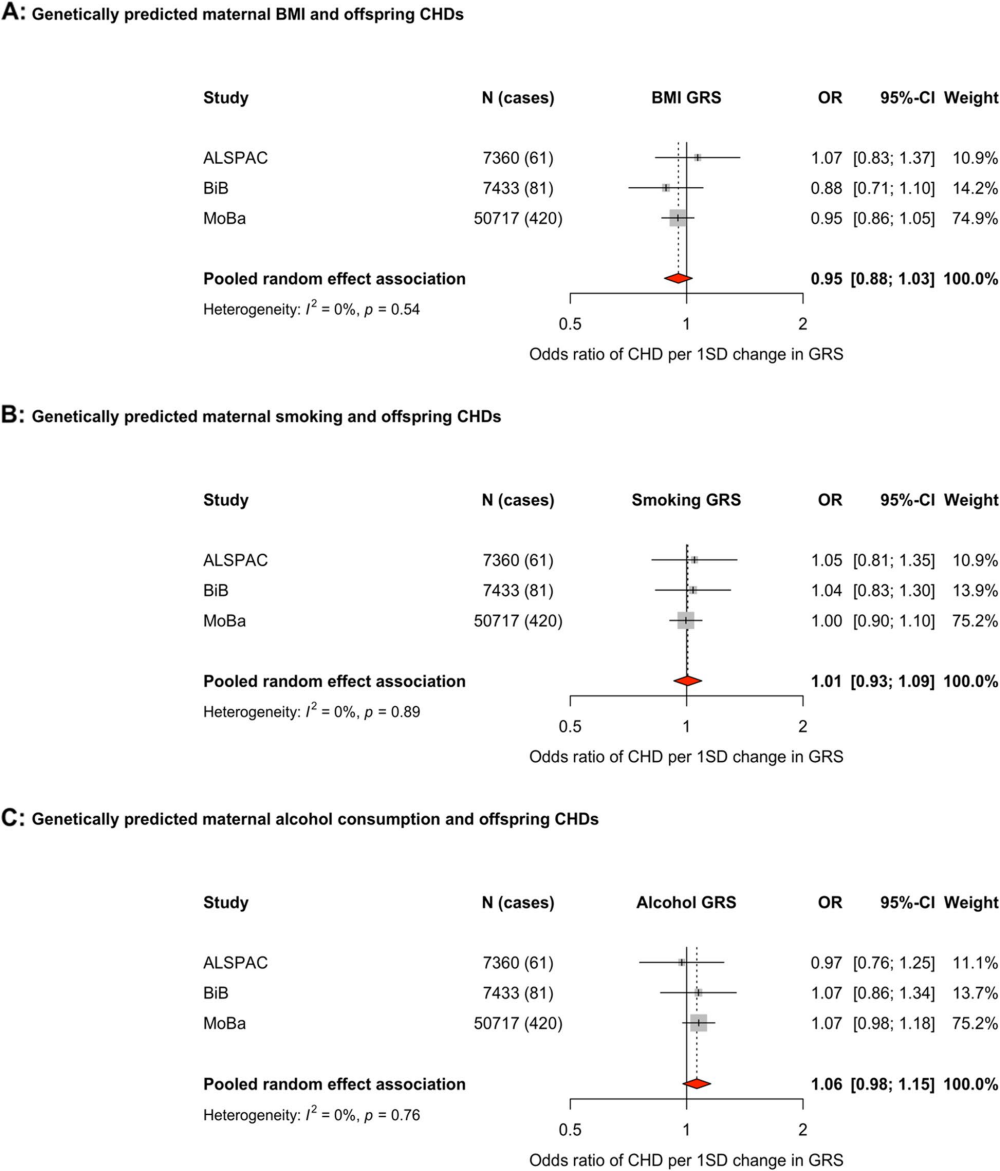
Forest plots showing the Mendelian randomisation results for genetically predicted maternal body mass index (**A**), any smoking (GRS generated using a GWAS of a lifetime smoking index: **B**), and any alcohol consumption (GRS generated using a GWAS of drinks per week: **C**) with offspring congenital heart disease. Odds ratios (ORs) of CHD for a 1SD difference in maternal GRS in each study and pooled across studies using random effects meta-analysis. Adjusted for top 10 genetic principal components in all cohorts with additional adjustment for genetic chip, genetic batch, and imputation batch in MoBa. Abbreviations: ALSPAC, Avon Longitudinal Study of Parents and Children; BiB, Born in Bradford; MoBa, Norwegian Mother, Father and Child Cohort Study; BMI, body mass index; CI, confidence interval; CHD, congenital heart disease; SD, standard deviation; GRS, genetic risk score

The MR effects in each study and pooled across studies of each exposure and offspring CHDs are shown in Fig. 2. For associations of the maternal GRS for BMI and offspring CHD, the pooled OR was below the null value of 1, with wide confidence intervals (CI) consistent with 12% lower to 3% higher odds (OR (95% CI) per 1SD higher GRS: 0.95 (0.88, 1.03), with no statistical evidence of between study heterogeneity (Fig. 2A). Results were unchanged when excluding BiB from these analyses (Additional File 2: Fig. S1B). The BMI GRS is associated with smoking, education, and diabetes across all three cohorts (Additional File 2: Table S3). Results were unchanged in MVMR models including GRSs for education and smoking (Figs. S1C and D). 47,970 participants with 376 CHD cases had data on foetal as well as maternal genotype. When the main maternal GRS association was undertaken in this subgroup, the result attenuated (OR: 0.84 (0.61, 1.15)). With additional adjustment for foetal genotype, the result was materially unchanged in the same subpopulation (OR: 0.83 (0.62, 1.11)). In subgroup analyses for those with foetal genotype data excluding BiB, the pooled results were more consistent and closer to the null (Figs. S1E–H).

The maternal GRS for maternal lifetime smoking index had a pooled OR close to the null, but with wide confidence intervals (OR (95%CI) per 1SD higher GRS: 1.01 (0.93, 1.09), with no statistical evidence of between study heterogeneity (Fig. 2B). The smoking GRS associated with BMI and education across the cohorts (Additional File 2: Table S4). Results were consistent and materially unchanged in additional analyses when removing BiB (Additional File 2: Fig. S2B) and in MVMR analyses adjusting for education or BMI (Figs. S2C and D). In the subgroup analyses with and without adjustment for foetal genotype, the point estimates increased slightly (Figs. S2E–H).

The maternal GRS proxying drinks per week also had a pooled OR close to the null, but with CIs ranging from 2% reduced odds to 15% increased odds (pooled OR: 1.06 (0.98, 1.15)) (Fig. 2C), with consistent associations found in BiB and MoBa. In analyses excluding BiB, the pooled estimated was consistent with main analyses (OR: 1.07 (0.97, 1.16)), although primarily being driven by MoBa. The alcohol GRS showed a consistent association with smoking across the cohorts (Additional File 2: Table S5). The results remained unchanged in MVMR analyses adjusting for a GRS of smoking (Additional File 2: Fig. S3C), but were attenuated closer to the null (with less precision) in analyses adjusting for offspring genotype (Figs. S3D–G).

## Discussion

Using MR across three birth cohorts, we found no strong evidence for an effect of genetically predicted maternal BMI, smoking or alcohol on risk of offspring CHD. However, for all three exposures, confidence intervals were wide and the pseudo *R*^2^ and AUC suggested potential weak instrument bias for alcohol and smoking. Weak instruments in this study would be expected to bias results toward the confounded association. Weak instruments and imprecise associations also limit clear interpretation of our sensitivity analyses to explore bias due to GRS influencing CHDs via other paths independently of the exposure of interest. We tried to identify all cohorts with maternal genetic data and offspring, CHD measures and to the best of our knowledge, this is the first MR study of these maternal exposures on offspring CHD risk. Despite the relatively large sample, our inconclusive findings highlight the importance of existing and new cohorts, many of which have genomic data, linking to health care records to obtain information on CHDs and other rare outcomes, for example through electronic health records.

This MR study complements our previous negative paternal control study [11]. Our MR analyses of BMI are consistent with our previous negative control study, in suggesting that higher maternal BMI may not causally influence offspring CHD and that previous multivariable regression analyses [6, 8] were likely confounded. We have not clearly replicated our previous result for smoking, which suggested an increased risk of offspring CHD in women who smoked in pregnancy. However, as noted above our imprecise MR results do not rule out an effect, and future larger MR studies are important. Due to the lack of information on alcohol consumption around the time of their partners pregnancy, previous analyses using a negative control design were inconclusive [11]. Recent meta‐analyses found consistently modest increases in risk of offspring CHD in mothers reporting alcohol consumption in pregnancy, however, many of the included studies did not adjust for confounders [10, 56], meaning that it is difficult to determine whether the association is a result of alcohol or other characteristics that are related to alcohol and offspring CHDs. In the present study, the results for alcohol were inconclusive, although notably confidence intervals of the pooled effect (pooled OR: 1.06 (0.98, 1.15)) did not rule out an association further emphasising the need for future larger studies.

There are several strengths of the current study. We attempted to identify all studies with relevant data knowing that MR is statistically inefficient, and CHD is a rare outcome. We explored potential bias due to other paths from the GRS to CHDs by examining associations of each GRS with the other two exposures and with other risk factors that might influence CHD and undertook multivariable MR where such associations were found. We also adjusted for offspring genotype in a subsample of the pooled data cohort, which is important in attempting to separate the influence of a path from GRS to CHDs via foetal genotype rather than solely from the mothers’ exposure [16].

The key limitation of this study is that despite a relatively large sample size (*N* = 65,510, *N* = 562 CHD cases) the effect estimates were imprecise due to CHD being a rare condition. In knowing this, we explored a collaborative base of birth cohorts and searched the literature for any additional cohorts that might contribute but found none that were eligible for inclusion. Furthermore, the GRS for smoking and alcohol may have been biased by the weak instruments. These limitations importantly contribute to our main and sensitivity analysis results. We were not able to clearly differentiate between horizontal (i.e., where smoking is the main exposure of interest, and an association of the smoking GRS with BMI reflects an independent path) and vertical pleiotropy (i.e. where the relation of the smoking GRS with BMI is downstream of the GRS effect on smoking). We want to adjust for horizontal pleiotropy but not vertical pleiotropy, as the latter would be adjusting away part of the mechanism by which, for example smoking might influence CHD. Adjustment for offspring genotypes was only possible in a subsample of the main analysis, making results more imprecise and prone to selection bias. However, it is encouraging that our results do not notably differ in these analyses. MR results may be biased by population stratification confounding. We tried to mitigate against that by adjusting for ancestry PCs and exploring the consistency of the main results with results removing BiB. Largely these were consistent but even more imprecise. Whilst we included all participants, including those from non-European ancestries, both MoBa (the largest contributing study) and ALSPAC participants are mostly of White European origin, and the GWAS data used to construct the GRSs were restricted to European participants. Therefore, our results may not generalise to other populations. By examining gene associations, without estimating causal effects, as we have done here (i.e. not adopting a formal IV framework), the three IV assumptions that we discuss need to be considered. However, with this approach, the fourth assumption, which is often ignored even in MR studies that do attempt to estimate causal effects, is not required. The fourth assumption which often receives less attention states that the effect of the exposure on the outcome may differ for different people [57, 58].

We were only able to explore associations of GRS with any CHD (analyses of subtypes would have been very imprecise) and therefore could have missed the potential effects of these exposures on specific CHD subtypes. Nevertheless, we believe there is value for prospective parents, clinicians and policy makers in knowing the effects of any CHD. MoBa cohort only had cases diagnosed antenatally or around the time of birth (first year of life) obtained from a single source (The Medical Birth Registry of Norway) which would increase the chances of outcome misclassification by assigning CHD cases which were diagnosed later in life as non-CHD cases. This misclassification is likely to be random with respect to the GRS (i.e., later age at offspring diagnosis could not influence mothers genotype) and would be expected to bias results towards the null, meaning we may have missed some associations. A previous study using MoBa data found a larger proportion of CHD cases than the present study (1.39% vs 0.83%) via ascertainment of linked health records [59]. We were unable to access these data for the present study. Potential reasons for the difference in birth prevalence could be firstly that they had access to more detailed ICD-coded data, and secondly because the MoBa genotype project datasets that we used in the present study had different inclusion criteria and therefore the two study populations are not directly comparable.

Identifying modifiable causal risk factors for the development of CHD is important for developing preventive interventions to reduce the risk of CHDs. Improvements in surgery over the last two decades mean that most patients with CHD now live into adulthood. Nonetheless, prevention remains important. Many patients require repeat procedures through childhood and adolescence to accommodate their growth, which produces a burden on them, their family and society. Despite trying to identify all relevant studies our results are inconclusive. They highlight the need for more data with maternal genetic and offspring CHD data. We think this is possible over the coming years as running GWAS is relatively cheap, and most cohort studies increasingly have these data. The following could considerably increase the sample size for MR in this field and result in key advances in preventing CHDs: (i) Add data on CHDs through electronic record linkage to existing cohorts; this was done recently in ALSPAC nearly 30 years after the original pregnancies [37]. Many of the cohorts that we considered for inclusion had genetic data but no information on CHD (or other congenital anomalies). (ii) Linkage to electronic records should be regularly updated at least until early adulthood so that cases that are diagnosed later in life are also captured [37, 38]. (iii) Ensure new cohorts, particularly large birth/pregnancy cohorts or those with the potential to prospectively collect data during pregnancy (such as the planned *UK Our Future Health*) gain consent to collect health data on CHDs (and other congenital anomalies). (iv) Continue to update the cohorts used in this study and update our results. For example, there are plans to continue running GWAS assays on mothers, fathers and offspring in MoBa who are currently not genotyped which will more than double the sample available in that study. (v) To the best of our knowledge, there are currently no publicly available GWAS summary statistics for CHD. To date, the largest GWAS for CHD in a European population included ~ 4000 cases [60]. As these GWAS continue to grow, significant data sharing and collaboration will be required, which could then pave way for large-scale two-sample MR studies to explore maternal risk factors for CHDs.

## Conclusions

The analysis steps taken in this paper aimed to explore the presence of a causal effect of maternal BMI, smoking and alcohol on offspring CHDs. In summary, we found no robust evidence of an effect for maternal genetically determined BMI or smoking on offspring CHD. We did observe a weak relationship between genetically predicted maternal alcohol intake on offspring CHDs, but this may be explained by weak instrument bias. Despite a large sample size, our results produced imprecise estimates. We have highlighted the need for future larger studies that employ a range of causal methods to further interrogate maternal gestational risk factors for offspring CHDs.

## Supplementary Information

**Additional file 1.** STROBE-MR checklist of recommended items to address in reports of Mendelian randomization studies.

**Additional file 2:** **Text S1.** Genetic data methods; Table S1. Further information on the genome-wide association studies used to generate genetic risk scores; **Text S2**. Genetic risk score generation; Text S3. Defining congenital heart disease; **Table S2**. Subcategories of CHD; Text S4. Describing the pregnancy phenotype data: maternal BMI, smoking, alcohol, education, parity, diabetes separated by each cohort; Text S5. Genetic risk scores for multivariable Mendelian randomisation (MVMR); **Table S3**. Exploring associations between the BMI GRS and risk factors for CHDs. We also include the association of the BMI GRS with BMI (also shown in Table 2 within the manuscript) for comparison; **Table S4**. Exploring associations between the smoking GRS (lifetime smoking index) and risk factors for CHDs. We also include the association of the smoking GRS with smoking (also shown in Table 2 within the manuscript) for comparison; **Table S5**. Exploring associations between the alcohol GRS (drinks per week) and risk factors for CHDs. We also include the association of the alcohol GRS with alcohol (also shown in Table 2 within the manuscript) for comparison; **Figure S1**. Showing the main results and results from additional analyses for the MR analyses of genetically predicted maternal BMI and offspring CHDs; **Figure S2.** Showing the main results and results from additional analyses for the MR analyses of genetically predicted maternal smoking (using a genetic risk score of a lifetime smoking index) and offspring CHDs; Figure S3. Showing the main results and results from additional analyses for the MR analyses of genetically predicted maternal alcohol consumption (using a genetic risk score of drinks per week) and offspring CHDs.

## Data Availability

The ALSPAC data management plan (http://www.bristol.ac.uk/alspac/researchers/data‐access/documents/alspac‐data‐management‐plan.pdf) describes in detail the policy on data sharing, which is through a system of managed open access. Scientists are encouraged to make use of the BiB study data, which are available through a system of managed open access. Please note that the study website contains details of all the data that is available through a fully searchable data dictionary and variable search tool" and reference the following webpage: http://www.bristol.ac.uk/alspac/researchers/our-data/. Before you contact BiB study, please make sure you have read the Guidance for Collaborators: https://borninbradford.nhs.uk/research/guidance-for-collaborators/). MoBa data are used by researchers and research groups at both the Norwegian Institute of Public Health and other research institutions nationally and internationally. The research must adhere to the aims of MoBa and the participants' given consent. All use of data and biological material from MoBa is subject to Norwegian legislation. More information can be found on the study website (https://www.fhi.no/en/studies/moba/for‐forskere‐artikler/research‐and‐data‐access/).

## Abbreviations

CHD: Congenital heart disease
BMI: Body mass index
MR: Mendelian randomisation
MoBa: Norwegian Mother, Father and Child Cohort Study
GRS: Genetic risk score
GWAS: Genome-wide association study
ALSPAC: Avon Longitudinal Study of Parents and Children
BiB: Born in Bradford
QC: Quality control
SNP: Single nucleotide polymorphism
MAF: Minor allele frequency
HWE: Hardy-Weinberg equilibrium
LD: Linkage disequilibrium
EUROCAT: European surveillance of congenital anomalies
MBRN: Medical birth registry of Norway
ICD-10: International classification of disease version 10
STROBE-MR: Strengthening the Reporting of Observational Studies in Epidemiology Using Mendelian Randomisation
OR: Odds ratio
SD: Standard deviation
PC: Principal component
CI: Confidence interval
MVMR: Multivariable Mendelian randomisation
